# Computational prediction of disease related lncRNAs using machine learning

**DOI:** 10.1038/s41598-023-27680-7

**Published:** 2023-01-16

**Authors:** Razia Khalid, Hammad Naveed, Zoya Khalid

**Affiliations:** 1 Computational Biology Research Lab, Department of Computer Science, National University of Computer and Emerging Sciences, NUCES-FAST, Islamabad, Pakistan; 2 National Center for Bioinformatics (NCB), Quaid-i-Azam University, Islamabad, Pakistan

**Keywords:** Computational biology and bioinformatics, Health care

## Abstract

Long non-coding RNAs (lncRNAs), which were once considered as transcriptional noise, are now in the limelight of current research. LncRNAs play a major role in regulating various biological processes such as imprinting, cell differentiation, and splicing. The mutations of lncRNAs are involved in various complex diseases. Identifying lncRNA-disease associations has gained a lot of attention as predicting it efficiently will lead towards better disease treatment. In this study, we have developed a machine learning model that predicts disease-related lncRNAs by combining sequence and structure-based features. The features were trained on SVM and Random Forest classifiers. We have compared our method with the state-of-the-art and obtained the highest F1 score of 76% on SVM classifier. Moreover, this study has overcome two serious limitations of the reported method which are lack of redundancy checking and implementation of oversampling for balancing the positive and negative class. Our method has achieved improved performance among machine learning models reported for lncRNA-disease associations. Combining multiple features together specifically lncRNAs sequence mutation has a significant contribution to the disease related lncRNA prediction.

## Introduction

Cell, the basic unit of life, has several ways to transform DNA into proteins. Generally, it goes through a two-step process that, first, transcribes DNA into RNA transcripts and then these transcripts are translated into proteins^1,2^. RNAs, therefore, are the result of DNA transcription. They are further classified into two groups; coding RNAs and non-coding RNAs. A messenger RNA (premRNA) or a coding RNA is a result of transcription which goes through an RNA splicing process wherein exons are joined together and introns are removed; and finally, proteins are produced during the process of translation^1^. On the contrary, a non-coding RNA (ncRNA) does not encode proteins but is used in other biological processes, particularly regulatory processes^3^.

According to the studies, less than 2% of the genome encodes proteins and the rest 98% genome is referred to as non-coding RNAs (ncRNAs)^4,5^. From these non-coding RNAs, 70% of the non-coding sequences are transcribed into long non-coding RNAs (lncRNAs). LncRNAs are usually greater than 200 nucleotides in length. LncRNAs have a significant role in many biological processes e.g., imprinting, immune response and cell differentiation^5,6^.

Many previous studies have reported the association between lncRNAs and diseases which involves the dysregulation of lncRNAs that contributed to the development of complex diseases such as cardiovascular diseases, Alzheimer’s, and different types of cancer^4,7^. According to the World Health Organization^8^ after heart diseases, cancer accounts for the most deaths worldwide. In 2018 alone, 9.6 million deaths were reported from cancer which accounts for one in every six deaths. Low and middle-income countries were particularly affected as 70% of the total deaths due to cancer were in these countries^8^. A cheap and easily scalable solution, thus, can directly help in curbing cancer mortality in these countries. Cancer is generally caused by mutation in various genes^7^. In earlier studies, it was examined that lncRNAs can act as both oncogenic and tumor suppressors. It is widely known that one of the major causes of cancer is abnormal cell growth which spreads or invades into other parts of the human body^4,5^. Mutation or overexpression of oncogenic lncRNAs in tumor cells disrupt oncogenic pathways leading to uncontrolled tumor development. On the contrary, tumor suppressor lncRNAs are among the major determining factors in cancer protection by promoting tumor suppressor pathways^7,9^. Two examples to illustrate this are HOTAIR^6,10^ and MALAT1 lncRNAs; wherein, overexpression of the former is the leading cause of breast cancer and the latter has a part in lung cancer development.

In recent years, a large amount of experimental data has been generated on the lncRNA-disease association. Several repositories have been created to store such amount of data e.g., CRLncRNA^11^, Lnc2cancer^12^, LncRNADisease^13^ and MNDR^14^. However, these experimental methods are very time consuming and expensive. To complement experimental methods, various computational approaches have been proposed lately, to identify the potential lncRNAs involved in disease formation. Based on this approach, the authors in^15–17^ built machine learning models to identify cancer-related lncRNA by exploiting the sequential features.

Zhao et al.^15^ introduced a model using Naive Bayes classifier by combining features from three categories; transcriptome, regulome and genome. Their positive data set consisted of 70 cancer-related LncRNAs and for the negative dataset, they used 205 lncRNAs. The reported AUC of the model is 0.793. Another study carried out by Zhang et al.^16^, built a cancer-related Long noncoding RNA classifier using four categories of features namely expression, genomic, network and epigenetic. They used five different machine learning classifiers i.e., Support Vector Machine, K-nearest Neighbors, Logistic Regression, Naive Bayes and Random Forest. Among those, the Random Forest was the best model among others in sensitivity and specificity. The reported AUC of the model is 0.82. They later updated their method to improve the accurate prediction of their model by using eXtreme Gradient Boosting (XGBoost) instead of Random Forest. In addition to that, they employed over-sampling and performed feature selection to improve their AUC to 0.86. The reported F1 score, precision and recall of the model are 0.65, 0.72, 0.62^17^.

Apart from sequential features, few studies^9,18^ have incorporated the pathways information for the identification of cancer-related LncRNAs. Chen et al.^18^ used KEGG pathways and GO ontology terms enrichment scores of genes that are co-expressed with lncRNA to identify cancer-related lncRNA. They analyzed RNA sequencing data of Illumina body map that include 16 normal human tissues to identify mRNAs that are affected by each lncRNAs. They considered those lncRNAs and protein-coding genes as co-expressed which had expressions greater than 0.7 according to Pearson coefficient correlation and these same co-expressed mRNAs were used to annotate the function of lncRNAs. They also measured the association of each lncRNA against each KEGG pathway and GO term and then they developed GO and KEGG enrichment scores as features. Feature selection and Dagging classifier (in which sequential minimal optimization was the basic classifier) were used. In the end total, 1468 optimal features were obtained with sensitivity, specificity, ACC and MCC equal 0.57, 0.96, 0.83, 0.62 respectively.

The major limitations found in the reported studies were the lack of redundancy checking on the sequence data. It is quite well known that if the sequences bear close similarity, then the model will not be able to generalize as there will be a possibility of sequence overlap between training and testing samples. Another drawback of these studies was using an insufficient positive dataset that may cause model underfitting. We, however, increased the positive data related to diseases for our study by extracting it from different sources. Keeping this in consideration, the aim of the current study is to develop a computational method based on machine learning that will integrate features derived from the sequence and structure of lncRNAs. Identification of cancerous and disease-related lncRNAs can help in understanding the molecular mechanisms of human diseases which, in turn, may help in the process of diagnosis, therapy, and forecasting of complex human diseases. Furthermore, this identification may lead to disease biomarkers and may be helpful in drug discovery. Furthermore, a low cost and easily scalable solution in cancer classification, such as ML-based solutions, can control cancer mortality in low and middle-income countries as they have the highest cancer mortality rate.

## Methods

**Figure 1:**
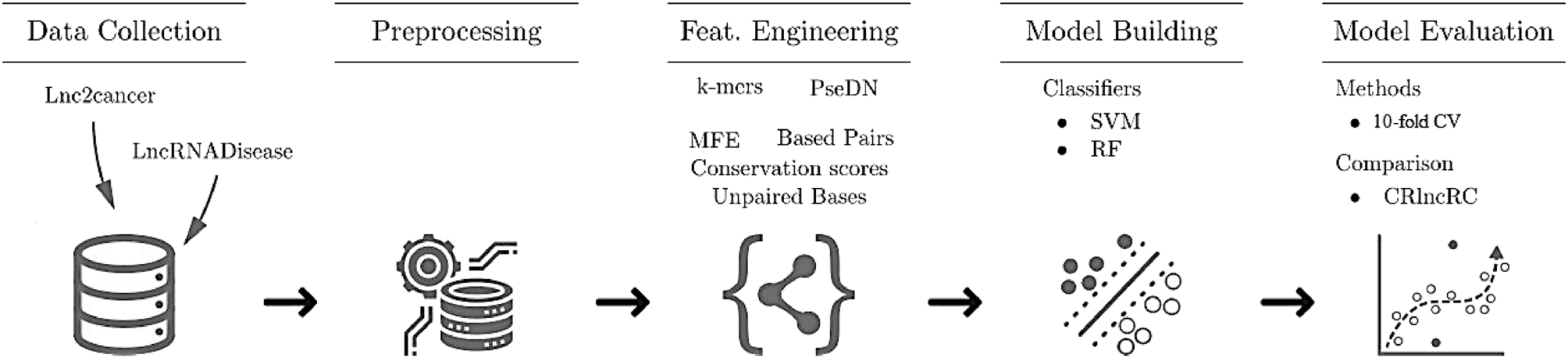
Workflow of methodology.

The Workflow of our methodology is shown in Fig. 1 consists of four major steps: data collection, feature engineering, model building, and model evaluation.

### Data collection

For this study, cancer-related and disease-related data were collected from two open-source databases Lnc2cancer^12^, and LncRNADisease^13^. After combining data from these sources, the overlapping data was removed and a dataset was built consisting of experimentally verified disease-related lncRNAs. For each lncRNA, we selected only those transcripts as disease-related that are mentioned in these two databases. As for negative samples, we used the dataset reported in Zhang et al.^17^. For this, we chose only the longest transcript from each lncRNA. We, then, annotated the data against each Ensemble transcript ID and its respective sequences, both of which were obtained from GENCODE v38^19^. In the end, we had a dataset of total of 689 disease associated LncRNA transcripts and 735 lncRNA transcripts as negative dataset. Table 1 shows the statistics of the data.

**Table 1:** Data statistics.

	Positive	Negative	Total
LncRNAs			1295
LncRNAs transcripts	689	735	1424
After 60% similarity			
LncRNAs			1204
LncRNAs Transcripts	524	690	1214

### Data pre-processing

During the pre-processing phase, we checked for redundancy between sequences and, for it, we performed sequence similarity on our whole dataset. We used the CD-HIT^20^ program for this task as it is used for the clustering and comparing of nucleotide sequences. We set the cut-off threshold at 60% and sequences with similarity ≥ 60% were removed. After this analysis, we were left with a total of 524 positive and 690 negative lncRNAs as tabulated in Table 1.

#### Feature extraction

To differentiate between disease-related and normal lncRNAs, we have extracted sequence-based and structure-based features. We combined important features from these categories and use them to build our model.

### Sequential features

Sequential features extracted from the RNA sequences are detailed below.

#### k-mers

K-mers are the subsequence of the length k in an RNA or DNA sequence. We extract k-mers from the sequence by running a k length window over all the sequences. A sequence of length L will have L—k + 1 k-mers and total n^k^ possible k-mers; where n is the number of possible nucleotides that are 4 in the case of RNA and DNA. We used k = 2, 3 and extracted 16 and 64 k-mers features from a sequence respectively.

#### Pseudo dinucleotide composition

The Pseudo Dinucleotide Composition (PseDNC)^21^ describes the contiguous local sequence-order information and the global sequence order information of lncRNAs. The pseudo dinucleotide composition has several variants and we used the parallel correlation pseudo dinucleotide composition (PC-PseDNC-General) which had the occurrences of different dinucleotides and their physicochemical properties. We used the Pse-in-One web server to extract the pseudo dinucleotide features^22^. We used the default parameter for lambda = 10, w = 0.05 and by choosing tilt, rise, roll, slide, twist and shift physiochemical properties, we obtained a feature vector of length 26.

#### Sequence conservation scores

*A* Conservation score is a score assigned to each nucleotide in a multiple species alignment to determine how conserved the nucleotide is. Conservation scores for each exon in LncRNA transcript were calculated on a per base basis using the phastCons 20way vertebrate genome alignment. The same was downloaded from the UCSC genome database^23^ (genome version GRCh38/hg38, genome annotation version GENCODE v38). We also took the mean of conservation score per exon and then the mean of exons per transcript to obtain a single feature.

#### GC content

GC content is the percentage of nitrogenous bases in DNA and RNA. We consider the GC content in exons of each transcript. The GC content was downloaded from the UCSC genome database^23^ (genome version GRCh38/hg38, genome annotation version GENCODE v38).

#### LncRNA-protein interactions

LncRNA-protein interaction data was downloaded from NPInter^24^ and starBase^25^. We counted the number of proteins interacting with LncRNAs and used this as a feature value.

#### Mutation count

Mutation in lncRNAs is one of the factors linked with disease association. Cosmic Noncoding Variant (cosmicNCV) data was downloaded from lncRNASNP2^26^ and LincSNP3.0^27^ databases. We counted the number of mutations in each LncRNA transcript and this count is added in the feature vector.

### Structural features

RNA structures are essential for understanding RNA function. Secondary structures are also considered important in playing multiple biological functions as they are more conserved than primary sequences. When a single-stranded molecule folds back on itself double-stranded RNA regions are formed which in turn form secondary structures^28,29^. For the computation of these secondary structures, the RNAfold python package by ViennaRNA was used which predicted the secondary structure of single-stranded RNA sequences^30^. This package returned the secondary structure in the dot-bracket notation; where parentheses were paired bases and dots were unpaired bases. The structural motifs in RNA secondary structures are classified as stems, hairpins, internal loops and multibranch loops^28,29^. The features that are extracted from structures are detailed below.

#### Minimum free energy

Minimum free energy shows the stability of RNA structure. The lower the energy is the more stable the structure will be. We used MFE obtained with structures from the RNAfold package as a feature.

- *Base pairs* Base pairs are considered the most stable in the structure. We counted the number of paired bases that included A-U, G-C, G-U, U-A, C-G, and U-G pairs in the structure as features.
- *Unpaired bases* We also counted the number of unpaired bases that included bulges, hairpins, and loops in structures as features.

#### Feature selection

Feature Selection is a Feature Reduction process used to reduce the number of input variables or features. The principal reason for it is to deal with the high-dimensionality problem which, if not taken care of, increases the training time of machine learning algorithms and often leads to overfitting of the model. The Feature Selection process chooses the most significant features from a large data set, which as a result improve the predictive model performance by removing the redundant and noisy features from the data. This makes the classifiers run faster and more effectively. We used the SelectFromModel method that is based on a Machine Learning Model estimation for selecting the features. We used Random Forest as an estimator with n_estimators = 50, max_depth = 11 and criteria = entropy. We have a total of 120 features and after feature selection, we were left with 25 features. The details of the features are listed in supplementary table S1.

### Model building

We have implemented SVM and Random Forest classifiers to classify disease-associated lncRNAs. We trained sequence and structure-based features separately and combined on both classifiers to check the model’s performance. Then using the grid search approach, we tuned hyperparameters of both classifiers to get the best possible parameters. For SVM the best performance was obtained using C = 0.1, gamma = 0.001, and kernel = linear. For the RF classifier, the best performance was obtained using n_estimators = 130, max_depth = 9 and criteria = entropy. We have performed stratified tenfold cross-validation on our data and to evaluate our model we used the F1 score which is regarded as the most appropriate evaluation measure for the imbalanced data.

Furthermore, we have also applied Extreme learning machine (ELM) algorithm to the dataset. We trained the ELM model only on selected features by performing tenfold cross-validation. We tuned the ELM with a different number of neurons in the hidden layer and chose the one which gave the highest F1-score. The highest F1-score was achieved using neurons = 22 in hidden layers and activation function = sigmoid.

## Results and discussion

To predict disease-related lncRNAs, machine learning classifiers SVM and Random Forest were used. To better understand the importance of each feature we have trained the models with all features separately and then combined to determine the contribution of each feature. The sequence features extracted from the LncRNAs sequence are k-mers (k = 2, 16-features; k = 3, 64-features;), PseDNC (26 features), Conservation scores (1-feature), GC content (1-feature), LncRNA-protein interactions (1-feature) and mutation count (1-feature). While the structure features are MFE (1-feature), Base paired (6-features) and Unpaired bases (3-features). A significant increase in the performance measure was observed when lncRNA sequence mutation was added as a feature. We have tested the model with and without mutation count feature and observed an increase in F-score 70 to 74 in case of Random Forest and from 71 to 75 in case of SVM. Since mutations has an important role in disease association, understanding the contribution of this particular feature will be helpful in identifying the disease mechanism. The detailed results are tabulated in supplementary Tables S2 and S3.

### Evaluating disease related lncRNAs

We used our pretrained SVM model to predict 816 unknowns LncRNA from TANRIC dataset. These lncRNAs were not in our training dataset. Our model predicts 362 LncRNAs transcript as diseases related which belong to 198 unique genes. To test our prediction, we used MNDR V3.1 as we did not collect our training data from this database. We checked the intersection of our prediction with their collection. Among our top 65 LncRNAs we found 40 were also experimentally validated by MNDR as disease related. In total 102 disease related lncRNAs were found in MNDR.

### Cross validation accuracy

**Figure 2:**
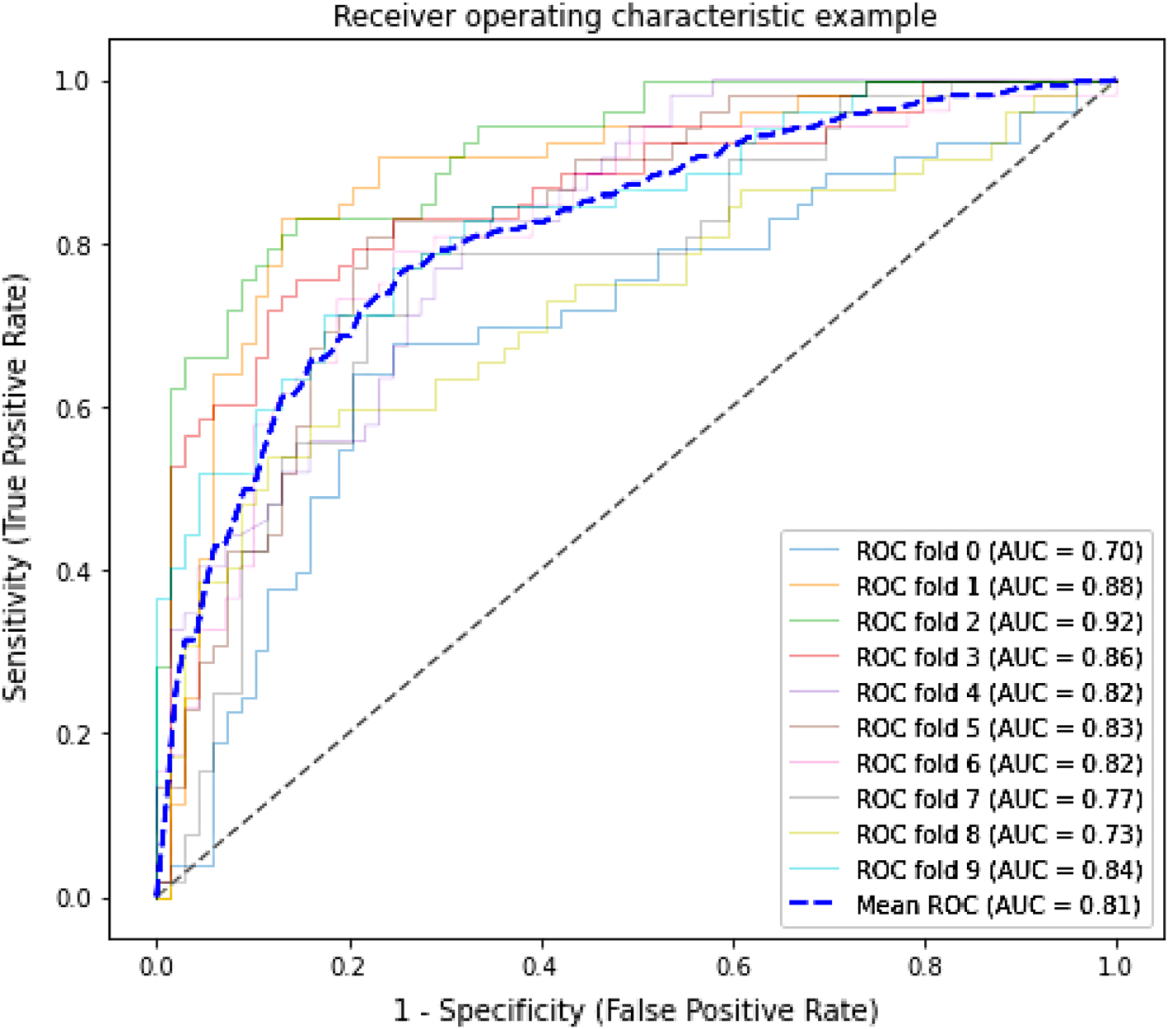
ROC curve for SVM on tenfold cross validation.

We trained our data using stratified tenfold cross-validation and evaluated our model performance with Accuracy, F1score, precision and recall. The data is divided into 10-folds, where each time 9-folds are used for the training set and one-fold used for the validation set. We trained a total of four models. The first two models were trained on sequence features and all features (sequence and structure features) for RF. The next two models were trained on sequence features and all features (sequence and structure features) for SVM. The results are tabulated in Table 2. We also performed feature selection using SelectFromModel. The results of SVM, RF and ELM after feature selection are tabulated in Table 3. Along with F1-score, precision and recall, we also evaluated our model with AUC. For this, by using tenfold cross validation, we also drew ROC curves, with maximum and minimum AUC values of 0.92 and 0.70, respectively, and average AUC value of 0.81 as shown in Fig. 2.

**Table 2:** Performance of SVM and random forest without feature selection using tenfold CV.

Models	Features	F1-score	Precision	Recall
RF	Sequence features	73	74	72
RF	All features	74	75	74
SVM	Sequence features	75	76	75
SVM	All features	75	76	75

**Table 3:** Performance of SVM, random forest and ELM with feature selection using tenfold CV.

Models	No. of features	F1-score	Precision	Recall
RF	25	76	76	75
SVM	25	76	77	75
ELM	25	73	73	72

### Comparison with the state-of-the-art

**Figure 3:**
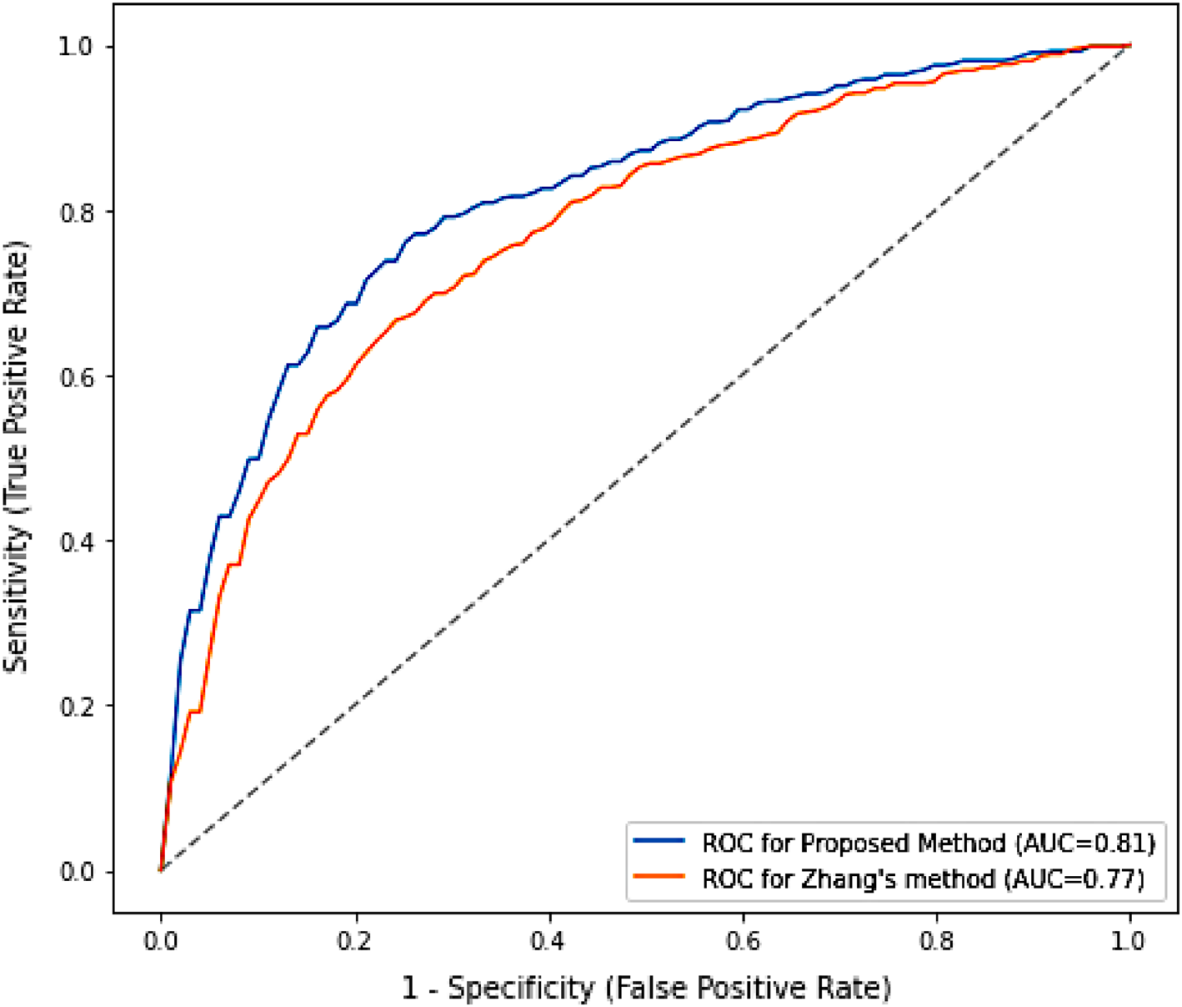
Comparison of the roc curves with the state-of-the-art.

For the comparison, we compared the prediction performance of our approach with the method described by CRlncRC2^16^. CRlncRC2 used an XGBoost classifier to predict disease-related lncRNAs by integrating genomic, epigenetic, network and expression features. To ensure a fair comparison, we trained our data on the CRlncRC2 method using stratified tenfold cross-validation. On the CRlncRC2 method, the model has achieved an accuracy of 72% with an average F1 score of 71%, precision of 72% and recall 71% which is better than the results in their study (F1-score 65%). The results are tabulated in Table 4. It is important to note that our method works on transcript level data to identify only those transcripts that are disease-related rather than taking the whole gene. In terms of AUC the proposed method has achieved higher AUC of 0.81 as compared to the state-of-the art method (AUC = 0.77). The results are figured in Fig. 3.

**Table 4:** Comparison with state-of-the-art.

Models	Accuracy	F1-score	Precision	Recall
Proposed method	77	76	77	75
CRlncRC2 Zhang et al.	72	71	72	71

## Conclusion

Complementing experimental approaches by computationally predicting lncRNAs-disease associations can make the predictions speedy and cost-effective. The current study developed an improved machine learning model that integrates multiple features to enhance its prediction performance. The results showed a significant improvement over the previous reported methods. Among the ML models, SVM has achieved the highest performance with an F1 score of 76%. To validate our approach, we also compared our results with state-of-the-art method. To the best of our knowledge, no previous studies have used this combination of features to predict disease related lncRNAs. Thus, our novel combination of features would be a significant addition in the literature for the future disease related lncRNAs prediction studies.

## Supplementary Information

Supplementary Information.

## Data Availability

The datasets used and/or analyzed during the current study available from the corresponding author on reasonable request.
