# Supplementary material for: Computational prediction of disease related lncRNAs using machine learning
Source: Sci Rep. 2023 Jan 16;13:806. doi: 10.1038/s41598-023-27680-7 (PMC9842610; doi:10.1038/s41598-023-27680-7)
Supplement: Supplementary file 1 — Supplementary Information. [file 41598_2023_27680_MOESM1_ESM.pdf]

**Sequence Features**							**Structure features**		
**Feature number before feature selection**									
2mers	3mers	PseDNC	Cons score	Gc content	lncrna-protein-interaction	mutation count	MFE	Paired bases	Unpaired bases
**16**	**64**	**26**	**1**	**1**	**1**	**1**	**1**	**6**	**3**
**Feature number after feature selection**									
**4**	**9**	**6**	**1**	**1**	**1**	**1**	**1**	**1**	**0**

Table S1: Number of features after feature selection

**Features**	**Accuracy**	**F1_macro**	**Precision**	**Recall**
2mers	69	68	69	68
3mers	70	69	70	69
PseDNC	67	65	67	65
structures	64	62	63	62
2mers,3mers, pse,cons score, gc content	71	69	71	69
2mers,3mers, pse,cons score, gc content, lncrna-protein-interaction	72	70	72	70
2mers,3mers, pse,cons score, gc content, lncrna-protein-interaction, structure	71	70	71	70
2mers,3mers, pse,cons score, gc content, lncrna-protein-interaction, mutation count, structure	75	74	75	74

Table S2: Result of individual features on Random Forest Model

Features	Accuracy	F1_macro	Precision	Recall
2mers	68	66	68	66
3mers	71	69	70	69
PseDNC	67	66	67	66
**structures**	64	61	64	62
2mers,3mers, pse,cons score, gc content	70	69	69	69
2mers,3mers, pse,cons score, gc content, lncrna-protein-interaction	72	71	71	71
2mers,3mers, pse,cons score, gc content, lncrna-protein-interaction, structure	72	71	72	71
2mers,3mers, pse,cons score, gc content, lncrna-protein-interaction, mutation count, structure	76	75	76	75

Table S3: Result of individual features on SVM Model
